# BMI-1 Autoantibody as a New Potential Biomarker for Cervical Carcinoma

**DOI:** 10.1371/journal.pone.0027804

**Published:** 2011-11-21

**Authors:** Yong-Qing Tong, Bei Liu, Hong-Yun Zheng, Yu-Juan He, Jian Gu, Feng Li, Yan Li

**Affiliations:** 1 Department of Clinical Laboratory, Renmin Hospital of Wuhan University, Wuhan, Hubei, People's Republic of China; 2 Clinical Molecular Diagnostic Center, Renmin Hospital of Wuhan University, Wuhan, Hubei, People's Republic of China; 3 Department of Pathology, Affiliated Tianyou Hospital of Wuhan University of Science and Technology, Wuhan, Hubei, People's Republic of China; University of Ottawa, Canada

## Abstract

BMI-1 is overexpressed in a variety of cancers, which can elicit an immune response leading to the induction of autoantibodies. However, BMI-1 autoantibody as a biomarker has seldom been studied with the exception of nasopharyngeal carcinoma. Whether BMI-1 autoantibodies can be used as a biomarker for cervical carcinoma is unclear. In this study,BMI-1 proteins were isolated by screening of a T7 phage cDNA library from mixed cervical carcinoma tissues. We analyzed BMI-1 autoantibody levels in serum samples from 67 patients with cervical carcinoma and 65 controls using ELISA and immunoblot. BMI-1 mRNA or protein levels were over-expressed in cervical carcinoma cell lines. Immunoblot results exhibited increased BMI-1 autoantibody levels in patient sera compared to normal sera. Additionally, the results for antibody affinity assay showed that there was no difference between cervical polyps and normal sera of BMI-1 autoantibody levels, but it was significantly greater in patient sera than that in normal controls (patient 0.827±0.043 and normal 0.445±0.023; *P*<0.001). What's more, the levels of BMI-1 autoantibody increased significantly at stage I (0.672±0.019) compared to normal sera (*P*<0.001), and levels of BMI-1 autoantibodies were increased gradually during the tumor progression (stage I 0.672±0.019; stage II 0.775 ±0.019; stage III 0.890 ±0.027; stage IV 1.043±0.041), which were significantly correlated with disease progression of cervical carcer (*P*<0.001). Statistical analyses using logistic regression and receiver operating characteristics (ROC) curves indicated that the BMI-1 autoantibody level can be used as a biomarker for cervical carcinoma (sensitivity 0.78 and specificity 0.76; AUC = 0.922). In conclusion, measuring BMI-1 autoantibody levels of patients with cervical cancer could have clinical prognostic value as well as a non-tissue specific biomarker for neoplasms expressing BMI-1.

## Introduction

Cervical cancanoma is much more deadly in developing countries than in developed countries [1]. Cervical cancer incidence rates have decreased significantly in developed countries, largely due to the early diagnosis of precancerous lesions and early treatment following detection [2]. Due to the relative inefficiency of cervical screening in developing countries, the incidence of cervical cancer was six times as high as that in developed countries [3]. Screening is the basic practice in cancer prevention for cervical cancer [4]. There are several alternative techniques for screening for pre-cancerous lesions for cervical cancer, including the Pap smear, visual inspection with acetic acid (VIA), human papilloma virus (HPV) DNA testing and combined Pap smear and VIA [3], [4]. As cervical cytology screening has become more prevalent, preinvasive lesions of the cervix are detected far more frequently than invasive cancers. Early detection can make a significant difference for the treatment outcome of cervical cancer [1]. HPV testing is more sensitive, but less specific than conventional cytology for detecting high-grade cervical intraepithelial neoplasia (CIN) [5]. HPV testing is less specific than cytology because many infections regress without developing high-grade lesions [6], [7]. There is therefore a need to identify strategies for increasing specificity with HPV DNA testing while maintaining its advantage in terms of sensitivity.

BMI-1 is a transcriptional repressor, which belongs to the polycomb group family [8] and was originally identified as an oncogene that cooperates with c-myc in the oncogenesis of mouse lymphomas. BMI-1-deficient mouse embryonic fibroblasts (MEF) overexpress INK4a/ARF locus–encoded genes, p16INK4a and p19ARF (mouse homologue of human p14ARF) and undergo premature senescence in culture [9], [10]. Proper function of this family is maintaining gene expression patterns during development. This gene plays a key role in the self-renewal of stem cells. It has been demonstrated that over-expression of BMI-1 occurs in a variety of cancers [11], [12], [13], including several types of leukemia and solid tumors such as non–small cell lung cancer, mantle cell lymphomas, colorectal cancer and prostate cancer suggesting a role in tumor cell growth and survival. Recently, BMI-1 over-expression has been identified as a marker of poor prognosis and metastasis in breast cancer, acute myeloid leukemia and neuroblastoma. Notably, BMI-1 is associated with both humoral and T-cell responses, suggesting that it represents a novel family of tumor-associated antigens (TAAs) that might be potential target for immunotherapy [14], [15].

New biomarkers, such as autoantibody signatures, may improve the early detection of cervical carcinoma. Therefore,in this study, we employed efficient methodologies to determine levels of BMI-1 autoantibodies in patient sera from a cDNA T7 phage display library constructed with mixed cervical carcinoma tissues. The immunogenic BMI-1 protein expression in recombinant phage was detected through immunochemistry and ELISA. We further evaluated the sensitivity and specificity of ELISA for predicting cervical carcinoma.

## Materials and Methods

### Cell culture

Cervical carcinoma cell lines (including HeLa, Caski and SiHa), and normal cervical cell line H8 were cultured in RPMI 1640 medium supplemented with 10% fetal bovine serum and antibiotics (100 U streptomycin/100 U penicillin) in a humidified atmosphere at 37°C with 5% CO_2_. BMI-1 positive cell line K562 derived from chronic myeloid leukemia (CML) was used for control. All cell lines were obtained from China Center for Type Culture Collection, Wuhan University.

### Ethics statement

The study was approved by the Medical Ethics Review Committee of Renmin Hospital, Wuhan University. All participants in this study were required to provide a written informed consent in accordance with Renmin Hospital of Wuhan University Ethics Committee; patients under supervision of a lawful caregiver if necessary.

### Patients and Sera Preparations

Following informed consent, a total of 77 samples of peripheral blood was obtained from individuals with histologically confirmed cervical carcinomas prior to treatment at the Department of Gynaecology and Obstetrics, Renmin Hospital, Wuhan University. 52 samples of peripheral blood obtained from individuals with chronic cervicits, and 39 samples of peripheral blood from individuals with CIN. 73 samples of normal sera were obtained from healthy blood donors. The majority of patients were stage (stages I: n = 25; stages II: n = 23; stages III: n = 18; stage IV: n = 11). Cervical carcimoma was biopsy-proven in all squamous cell carcinoma types. Their ages ranged from 21 to 76 years, with a median age of 53 years. Clinicopathological parameters, such as the depth of tumor invasion, lymphatic and venous involvement, and regional lymph node metastasis were evaluated according to the International Federation of Gynecology and Obstetrics (FIGO) recommendation [16]. All samples were centrifugated for 5 min at 3000 g, and then frozen and stored at -80°C. Ten cervical carcinoma sera and 8 normal sera were used to biopan, and 67 cervical carcinoma samples (Table 1) and 65 normal samples, not including the sera used to biopan, were used to evaluate predictive value by ROC curves.

**Table 1:** Clinical and Pathological Information for Cervical Carcinoma Patients for ELISA.

Category	Subcategory	No. of patients
Age(years)		51±5.8
Tumor size	≤4cm	35
	>4cm	32
Clinical stage	I	23
	II	20
	III	15
	IV	9
Lymph node metastasis	Negative	38
	Positive	29
Recurrent	Negative	53
	Positive	14
Distant metastasis	Negative	51
	Positive	16

### RNA extraction and Reverse transcription-PCR (RT-PCR)

Total RNA was isolated from the cells by the acid guanidinium thiocyanate-phenol-chloroform method using a Trizol reagent (MBI Fermentas, Lithuania) according to the manufacturer's instructions. The RNA was treated with DNase, and 2.5μg of total RNA was utilized for cDNA synthesis using random hexamers (MBI Fermentas, Lithuania). Full-length open reading frame of BMI-1 was amplified by PCR from cDNA samples of cervical carcinoma cell lines. The following primers were used for amplification of BMI-1: sense primer, 5′-ACCTGATGTGTGTGCTTTGTG-3′; antisense primer, 5′-TGCTGGGCATCGTAAGTATCT-3′. The primers used for glyceraldehyde-3-phosphate dehydrogenase (GADPH, internal control) were 5′-AATCCCATCACCATCTTCCA-3′ and 5′-CCTGCTTCACCACCTTCTTG-3′. The PCR products were analyzed by agarose gel electrophoresis and confirmed by appropriate size and/or sequencing.

### Protein extraction and Western blot

Cells were washed with PBS and lysed on ice with cold RIPA buffer (Sigma-Aldrich, Steinheim, Germany) containing protease inhibitors (Complete Mini, Roche Diagnostics, Mannheim, Germany). Whole cell protein extracts were separated on 10% SDS-PAGE and electroblotted onto a nitrocellulose membrane (Amersham). The blocked membrane was incubated first with cervical carcinoma patient serum or Anti-β-actin antibody (Santa Cruz Biotechnology, Inc) and then with goat anti-human antibody conjugated with horseradish peroxidase (Promega). The signal was detected using Pierce ECL Western Blotting Substrate.

### Immunodetection to define differences in antibody reactivity of normal and cervical carcinoma patient serum

Using preferential affinity of antibodies found in cervical carcinoma patient sera and not in normal sera, BMI-1-expressing phages were isolated from a mixture cervical carcinoma tissues T7 phage cDNA library. Isolated phages expressing BMI-1 was plaqued to limiting dilution on LB-Agar/agarose plates. Individual protein-expressing phages were plaqued to limiting dilution on LB-Agar/agarose plates and then lifted onto a nitrocellulose membrane for immunodetection as above. The plaque-lifted membranes were cut into several strips and blocked with 5% blocking reagent in 1×TBST. The individual strips were incubated with serum from individual cervical carcinoma patients or pooled normal sera (1∶100) that were not used in the biopan procedure. Next, the strips were incubated with anti-human HRP-conjugated secondary Ab and detected with DAB staining as previously described [17].

### Enzyme-linked immunosorbent assay for antibodies against BMI-1

ELISAs were developed for the phage-expressed proteins to confirm their immunogenic reactivity with different patient serum. 96-well microtiter plates (Costar, Corning, USA) were coated with PEG-purified phages expressing BMI-1 or empty phages as a negative control (10^9^ phage/well in 1×PBS/1% BSA at 4°C, overnight), then blocked (PBS/BSA 37°C, 1h) and washed (PBS/Tween 20). Serially diluted (1∶20 to 1∶2560) serum samples from individual patients that were not used in the biopan were added to each well (37°C, 1h). The plates were washed (3 times, 5 min/time), and then incubated with anti-human HRP secondary Ab (37°C, 1h). Assays were developed with 2, 2′-azino-bis-(3-ethylbenzothiazoline-6-sulfonic acid) (ABTS)/H_2_O_2_ substrate (Sigma, German) for 20 min, and then read on a spectrophotometer at 405nm. Each individual serum was run in triplicate. In separated experiments sera were assayed at a single dilution (1∶100) and absorbance was used as a measure of antibody reactivity in each independent assay. 65 normal serum samples and 40 patient samples were assessed for antibodies to the phages expressing BMI-1. The data were analyzed for each marker and combinations of two or more markers.

### Statistical analysis

Results were expressed as mean ± Standard deviation (SD). Associations between different variables were assessed by the Mann–Whitney test and one-way analysis of variance (ANOVA) was used to compare continuous variables among the groups. Logistic regression was used to determine whether a sample was from a cervical carcinoma or a healthy donor. A model was constructed using BMI-1 autoantibody measurements as explanatory variable. Receiver operating characteristics (ROC) curves was used to evaluate the sensitivity and specificity for predicting cervical carcinoma. Statistical analysis was carried out using SPSS software, version 13.0 (SPSS Inc., Chicago,IL, USA). A *P* value less than 0.05 was considered statistically significant. All data were analyzed anonymously.

## Results

### The expression of BMI-1 levels in cervical carcinoma cell lines

**Figure 1:**
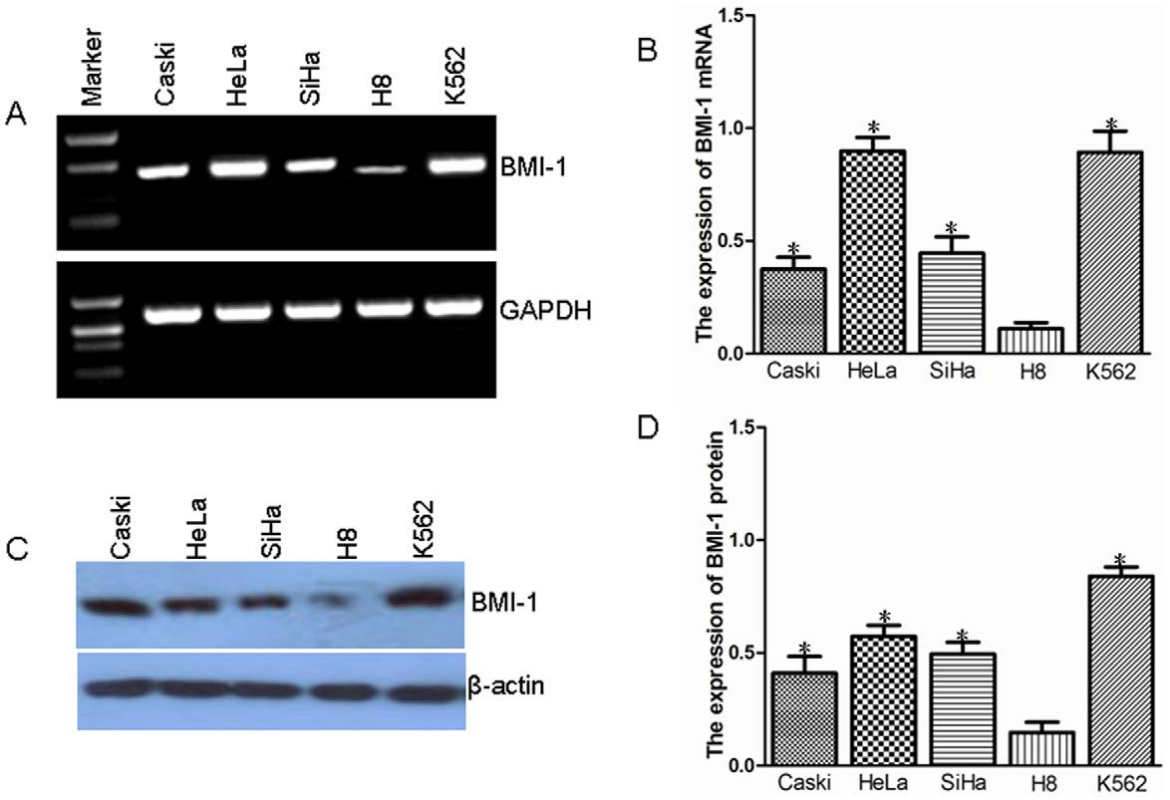
The expression of BMI-1 in cervical carcinoma cell lines and controls. A. RT-PCR analysis of BMI-1 mRNA in various cervical carcinoma cell lines. B. The ratio of BMI-1 mRNA expression in various cervical carcinoma cell lines. C. Immunoblotting of BMI-1 protein in various cervical carcinoma cell lines. D. Quantification of BMI-1 protein expression in various cervical carcinoma cell lines. The cervical carcinoma cell lines included HeLa, Caski, and SiHa. K562 was used as a positive control. H8 was a normal cervical cell line. GAPDH was used as an internal control.

We analyzed the expression of BMI-1 in several human cervical carcinoma cell lines using RT-PCR or Western blot. BMI-1 was expressed at different degree compared with the expression of control cell line (Fig. 1A and 1C). The ratio of BMI-1 expression in cervical carcinoma cell lines was singnificantly increased in all of the three cervical carcinoma cell lines compare to that in H8 cell line (Fig. 1B and 1D) (*P*<0.05).

### BMI-1 phage from a T7 phage cDNA library of mixed cervical carcinoma tissues reacted with patient's sera

**Figure 2:**
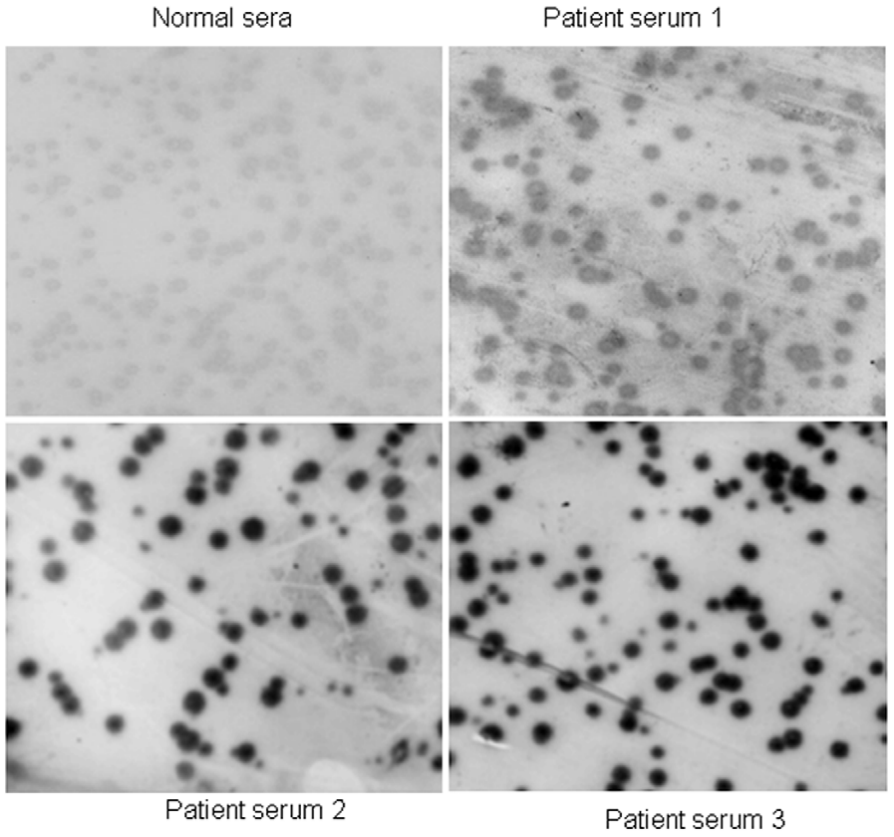
Immunodetection of BMI-1 expressing phages with cervical carcinoma patient and normal sera. Immunodetection was performed by DAB to define differences in antibody reactivity between normal and cervical carcinoma patient serum. T7 phage expressing BMI-1 was plated on LB-Agar and plaque lifted onto nitrocellulose membrane that was then cut into strips. Strips were incubated with pooled cervical carcinoma patient sera or normal sera followed by incubation with anti-human secondary antibodies. The sera were those used in the initial biopanning. Spots represent individual phage colonies. Comparisons between normal and cervical carcinoma sera for BMI-1 phage were shown.

Following a series of sequential selection and enrichment steps, we isolated phages that had high immunochemical reactivity with patient sera. After PCR amplification and sequencing, by comparison to known cDNA sequences in GeneBank, we identified BMI-1 from T7 cDNA phage library of mixed cervical carcinoma tissues. Relative specificity for this protein using this approach was confirmed by the comparison of immunochemical reactivity of normal and cervical carcinoma patient sera (Fig. 2).

### Antibody affinity for phage-expressing protein

**Figure 3:**
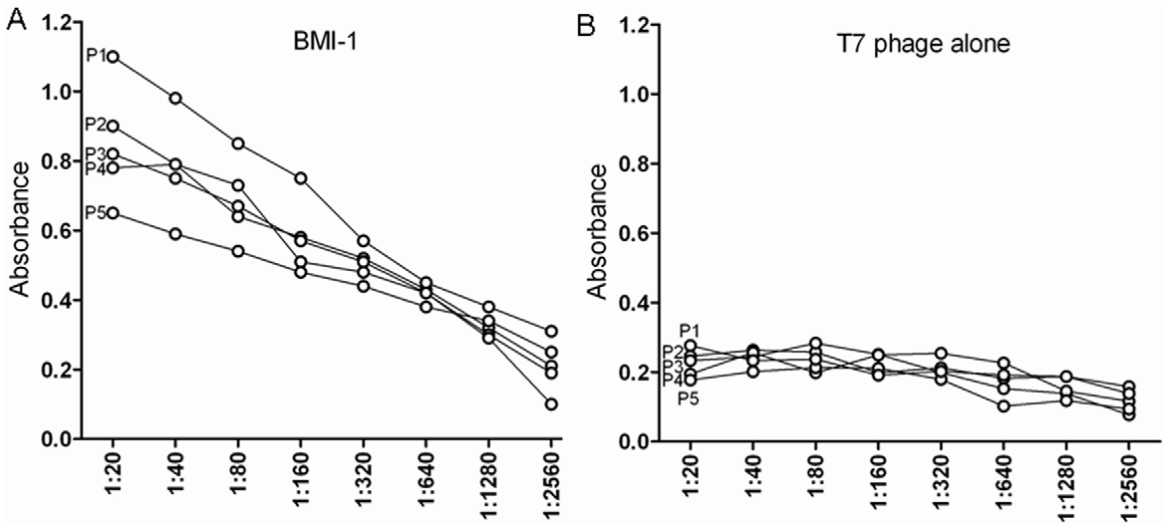
ELISA of phage-expressing BMI-1 proteins with individual serum samples. The assays were performed with serially diluted (1∶20 to 1∶2560) individual serum samples that were not used in the biopan to confirm that measurements were representative of an antigen-antibody affinity reaction. Representative curves from four patients (P1, P2, P3, P4, P5) are shown for each protein. Measurements are expressed as mean absorbance minus the value of each serum sample assayed against empty T7 phage (background) 6 SEM absorbance. Data from empty T7 phage at each dilution, for each patient sample, are shown as a reference.

To confirm antibody affinity in individual serum samples for specific protein, serum was assayed in limiting dilution from 1∶20 to 1∶2560 by ELISA constructed with phages BMI-1 and empty phages as control. Absorbance values for the antibody in sera of three patients decreased over serial dilutions, suggesting antibody affinity for BMI-1 protein (Fig. 3A). Empty phage controls exhibited background signals similar to negative controls in each ELISA (Fig. 3B).

### ELISA for antibodies against phage-expressing protein

**Figure 4:**
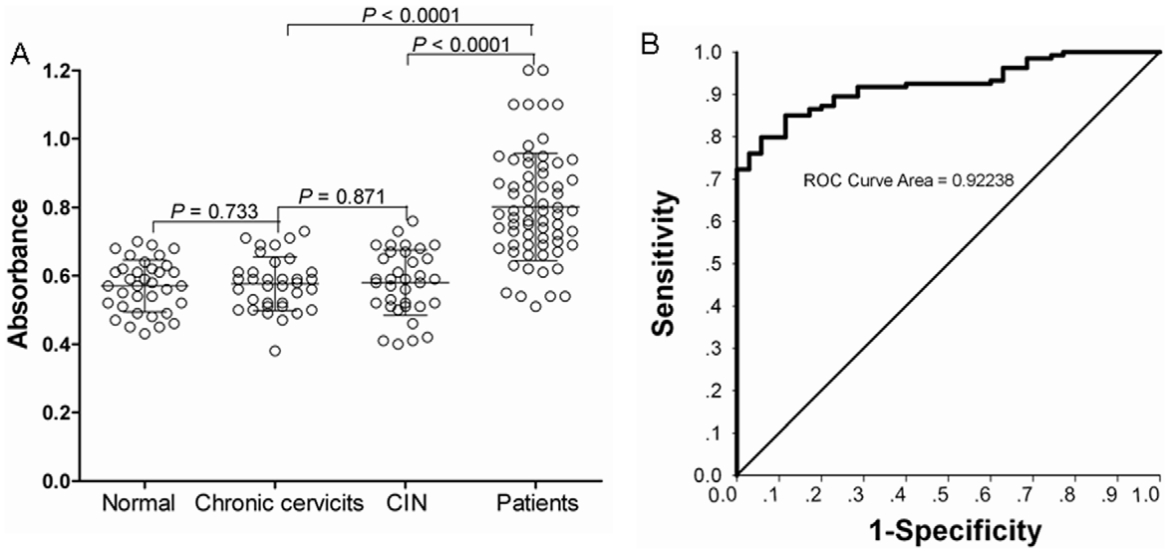
Comparative measurements of BMI-1 autoantibody in normal and cervical carcinoma sera. A. Box plots of measurement results of BMI-1 antibody for 65 normal and 67 cervical carcinoma patient sera using Elisa. Mean is shown by horizontal bar inside the box and the two horizontal bars above and below the box corresponding to mean ± 2 SD. The observations that fall outside 2 SD. from the mean are also shown in each plot. Mean ± SEM. for each are as follows: BMI-1: normal 0.570±0.013, cancer: 0.801±0.019; Values are expressed as absorbance. B. Data from quantitative ELISA for BMI-1 were evaluated for ability to predict disease. The lower black curve represents the predictive accuracy using the logistic regression model with BMI-1 ELISA data from 67 patients and 65 normal controls. The upper gray curve represents the predictive accuracy with BMI-1 as explanatory variable where *P*<0.0001 with c statistic 0.9224.

Sera from 65 normal and 67 cervical carcinoma patients were then evaluated to establish relative ranges for BMI-1 antibodies. The average of BMI-1 antibody absorbance values were significantly elevated above the mean of normal sera (0.570±0.0128 versus 0.801±0.019; P<0.001), and there were no significant differences between normal sera and the sera of chronic cervitics (0.576±0.013) and CIN (0.580±0.016) (Fig. 4A). Logistic regression was used to estimate the probability of a serum sample from a cervical carcinoma patient. Based on our data, it seems that such a model when using BMI-1 autoantibody measurements as an explanatory variable is highly reliable (*P*<0.0001). The ROC curve has a c-statistic (i.e. area under the curve or AUC-ROC) of 0.9224 with an optimal predictive accuracy of sensitivity 0.78 and specificity 0.76. (Fig. 4B).

### Correlation of levels of autoantibodies with clinical progression

**Figure 5:**
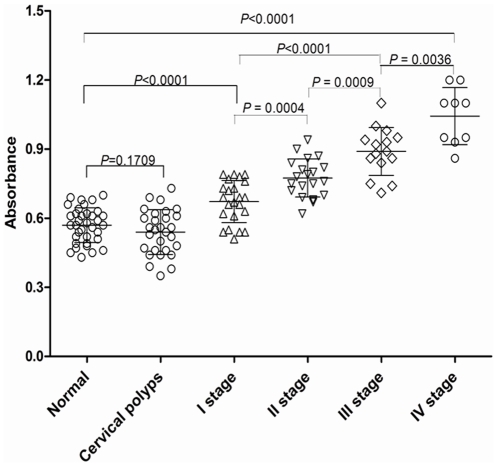
Measurement of levels of BMI-1 autoantibodies in normal, nasal polyp and cervical carcinoma sera. Plots of levels of autoantibodies from the sera of 65 healthy donors and 67 cervical carcinoma patients measured through Elisa. Mean is shown by the horizontal bar inside the box and the two horizontal bars above and below the box represent ±2SD. The observations that fall outside 2SD from the mean are also shown in each plot. Values are expressed as absorbance.

We further analyzed the relationship between BMI-1 autoantibody levels and clinical characteristics of patients. levels of BMI-1 autoantibodies were 0.570±0.013 in normal sera and 0.502±0.020 in cervical polyps, showing that there was no difference between them. The level of BMI-1 autoantibody increased significantly at stage I (0.672±0.019) compared to cervical polyps or normal sera (*P*<0.001). Moreover, the level of BMI-1 autoantibody kept increasing from stage I to stage IV, and the level of BMI-1 autoantibody was 0.672±0.019, 0.775±0.019, 0.890±0.027 and 1.043±0.041, respectively. Taken together, there was a significant correlation between BMI-1 autoantibody levels and clinical stages in cervical carcinoma group (*P*<0.001) (Fig. 5).

## Discussion

Despite the decrease in cervical cancer incidence due to improved diagnostic techniques and treatments, there is still a high mortality rate [2], [18]. A number of studies indicate that one or more oncogenic types of HPV may be responsible for cervical cancer, which is supported by strong epidemiological evidence and the detection of HPV DNA in 90–100% of cervical cancers [19]. HPV 16 and 18 are detected in 57.4% and 16.6%, respectively, of cervical tumor specimens [20], [21], [22], [23]. However, molecular mechanisms underlying the development and progression of cervical cancer remain poorly understood.

Currently, polycomb group (PcG) genes, which are pivotal in regulating gene expression through promoting chromatin modifications, have been deregulated in various human cancers [24], [25]. Therefore, deregulation of mammalian PcG members may contribute to human carcinogenesis. The transcription of two tumor suppressors, p16INK4a and p14ARF, human telomerase reverse transcriptase (h-TERT), as well as oncoprotein c-Myc, which have been implicated in the regulation of the cell cycle and proliferation, was found to be mediated by PcG proteins, mainly BMI-1, in vivo and in vitro. The BMI-1 was a known suppressor of p16 [26], [27]. The overexpression of p16 has been shown to be associated with progression to CIN3 or cervical carcinoma. HPV-positive women were also positive for p16 overexpression in cervical carcinoma [13]. Given the role of PcG proteins in epigenetic modification, it is possible that abnormal expression of PcG proteins contributes to the formation of cancer-specific cellular characteristics [28]. Recently, BMI-1, a member of the PcG group, has been found to be over-expressed in a variety of human cancers, including human cervical carcinoma [25], [29], [30] resulting in the induction of auto-antibodies that our studies indicate may be clinical useful. However, there was no evidence to show that the BMI-1 autoantibody exists in cervical carcinoma.

In our study, we identified BMI-1 from a T7 phage cDNA library of mixed cervical carcinoma tissues. Using ELISA and immunodetection approaches, we demonstrate for the first time that BMI-1 mRNA was over-expressed at different levels in cervical carcinoma cell lines. Moreover, antibody affinity assay using sera from 67 cervical carcinoma patients and 65 controls showed that the amount of BMI-1 antibody was significantly greater in patient sera than in normal controls (*P*<0.001). Levels of BMI-1 autoantibody were also significantly correlated with disease progression (*P* = 0.001), suggesting that BMI-1 could be a new candidate for screening test in cervical cancer. We further analyzed our results using logistic regression and ROC curve, which showed that the BMI-1 autoantibody as potential biomarker for cervical carcinoma has similar sensitivity and specificity to BMI-1. Although, the defined c-statistic or area under the ROC curve (AUC-ROC) of 0.9224 in our series indicates that the predictive value of BMI-1 autoantibody in cervical carcinoma is modest, cervical carcinoma heterogeneity showed similar limitations for other cervical carcinoma markers, such as CYFRA21.1, SCC and CEA, each varying somewhat with stage and histology [31], [32].

Over the years, numerous studies have revealed that the most probable order of events leading to cervical cancer formation is from normal cervical mucosa including acquisition of HPV infection, chronic cervicitis, followed by CIN [33], [34]. During such processes, a series of genetic mutations and deregulation in cellular homeostasis increase the likelihood of uncontrolled cell growth and malignancy [35]. However, in the present study, we did not admit CIN subjects and failed to compare the different expression in CIN patients and cervical cancer suffers, which would be our further experimental work.

In conclusion, we highlight the significance of BMI-1 autoantibody in cervical cancer. We showed that patients exhibiting higher BMI-1 autoantibody levels are associated with cervical carcinoma progression. Hence, detection of BMI-1 autoantibody levels may be of great clinical value to screen those with high risk of cervical cancer and BMI-1 autoantibody status may be useful to stratify patients for novel therapeutic strategies.
